# Successful paclitaxel-based chemotherapy for an alpha-fetoprotein-producing gastric cancer patient with multiple liver metastases

**DOI:** 10.1186/1477-7819-5-79

**Published:** 2007-07-16

**Authors:** Hiromitsu Takeyama, Hirozumi Sawai, Takehiro Wakasugi, Hiroki Takahashi, Yoichi Matsuo, Nobuo Ochi, Akira Yasuda, Mikinori Sato, Yuji Okada, Hitoshi Funahashi, Yoshimi Akamo, Tadao Manabe

**Affiliations:** 1 Department of Gastroenterological Surgery, Nagoya City University Graduate School of Medical Sciences, Kawasumi 1, Mizuho-cho, Mizuho-ku, Nagoya 4678601, Japan

## Abstract

**Background:**

Alpha-fetoprotein (AFP)-producing gastric cancer is known to frequently cause multiple liver metastases and to have an extremely poor prognosis.

**Case presentation:**

A 64-year-old Japanese man admitted to our hospital was diagnosed with gastric cancer with liver metastases. He underwent a total gastrectomy with splenectomy, and pathological stage IV disease according to the classification proposed by the Japanese Gastric Cancer Association was assigned. The histological diagnosis was poorly differentiated adenocarcinoma, and tumor production of AFP was confirmed by immunohistochemical staining. Following surgery, the patient received combination chemotherapy consisting of TS-1 and paclitaxel. Initially, AFP levels decreased dramatically and computed tomography (CT) revealed regression of liver metastases. However, multiple new liver metastases appeared and serum AFP levels increased after 5 months. A regimen of 5-FU plus paclitaxel followed by paclitaxel monotherapy was used next. Serum AFP levels once again decreased and CT showed regression or disappearance of liver metastases. The patient currently has a very good quality of life, and is receiving weekly paclitaxel monotherapy as an outpatient. No progression of liver metastases has been observed to date.

**Conclusion:**

We consider this rare case to have significant value with respect to treatment of AFP-producing gastric cancer with multiple liver metastases, and propose that combining surgery with chemotherapeutic agents such as paclitaxel may lead to a better prognosis in such cases.

## Background

Alpha-fetoprotein (AFP), which was initially identified from human fetal tissue, is normally produced in the fetal liver and yolk sac [1]. Since Bourreille *et al., *first reported a patient with gastric tumors that produced AFP, a considerable number of such patients have been identified [2]. AFP-producing gastric cancer is known to frequently cause multiple liver metastases and to have an extremely poor prognosis [3-6]. It has been reported that AFP-producing gastric cancer has high proliferative activity, weak apoptotic activity, and rich neovascularization compared with AFP-negative gastric cancer [4]. It is likely that these biological observations reflect the aggressive clinical behavior of AFP-producing gastric cancers.

There is no standard chemotherapy available for this disease, although the following regimens have demonstrated efficacy in a small number of cases: EAP (etoposide [ETP], adriamycin, and cisplatin [CDDP]), FAP (5-fluorouracil [5-FU], epirubicin [EPI], and CDDP), and FAP (5-FU, adriamycin, and CDDP) [7-9]. The therapeutic efficacy of irinotecan hydrochloride (CPT-11) and paclitaxel [10,11] in this disease state has also been recently reported.

In this report, we describe a case with AFP-producing gastric cancer that responded to combination 5-FU/paclitaxel chemotherapy followed by a bi-weekly course of paclitaxel monotherapy.

## Case presentation

**Figure 1:**
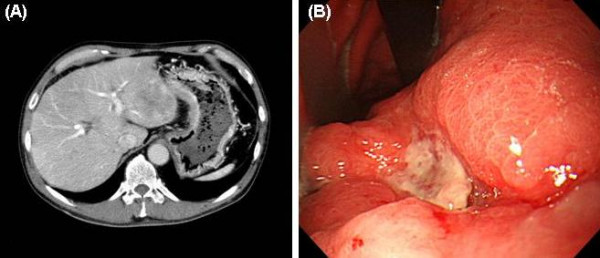
**(A) **Abdominal computed tomography (CT) revealed enhanced thickness of the gastric wall and multiple liver metastases. **(B) **Gastroscopy revealed a Borrmann type III tumor on the lesser curvature in the midportion of the stomach.

**Figure 2:**
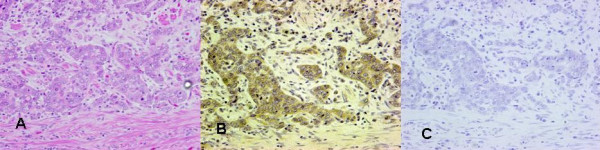
Resected specimen of stomach was fixed with 10% formalin and embedded in paraffin. Sections were stained with **(A) **hematoxylin-eosin staining (× 200) , **(B) **alpha-fetoprotein (× 200), or **(C) **negative control IgG (× 200).

A 64-year-old Japanese man admitted to Nagoya City University Hospital because of upper abdominal pain was diagnosed with gastric cancer with liver metastases. His family history was unremarkable. Laboratory data on admission revealed liver dysfunction as follows: serum glutamic oxaloacetic transaminase (SGOT), 125 U/L (normal range, 10–33 U/L); glutamic pyruvic transferase (SGPT), 252 U/L (normal range, 6–37 U/L); γ-glutamyl transpeptidase (γ-GTP), 1435 U/L (normal range, 10–47 U/L); alkaliphosphatase (ALP), 988 U/L (normal range, 115–359 U/L). AFP and carcinoembryonic antigen (CEA) levels were 1497.8 ng/ml and 72.7 ng/ml, respectively. Abdominal computed tomography (CT) showed enhanced thickness of the gastric wall and multiple liver metastases (Figure 1A). Gastroscopy revealed a Borrmann type III tumor on the lesser curvature in the midportion of the stomach (Figure 1B), which was diagnosed as gastric carcinoma upon histological examination of a biopsy specimen. After informed consent with enough detailed explanation about the patient's disease, he wished a surgical treatment strongly. The patient underwent surgical resection (total gastrectomy with splenectomy). The pathological stage was IV: T3, N1, H1, P0 according to the classification proposed by the Japanese Gastric Cancer Association [12], and the histological diagnosis was poorly differentiated adenocarcinoma. Lymphatic invasion was moderate and venous invasion was negative. Tumor production of AFP was confirmed by immunohistochemical staining (Figure 2A and 2B).

**Figure 3:**
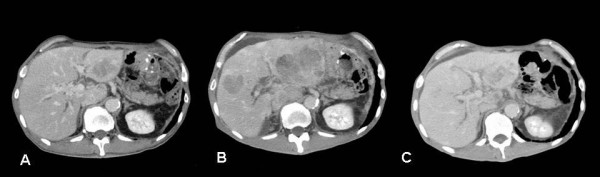
**(A) **Enhanced CT revealed that liver metastases had decreased in size following initial therapy. **(B) **After 5 months of treatment, multiple liver metastases reappeared in segments 1, 3, and 5. (C) Following second-line treatment with 5-FU/paclitaxel and paclitaxel monotherapy, liver metastases either regressed or disappeared.

The patient was treated with one course of paclitaxel (80 mg on days 1 and 8) and TS-1 (100 mg/day for 2 weeks and discontinuation for 1 week) after surgery. After 3 months, the level of AFP had remarkably decreased from 4344.0 ng/ml to 418.9 ng/ml. After 4 months, CT revealed regression of liver metastases (Figure 3A). However, after 5 months, multiple new liver metastases were apparent (Figure 3B) and serum AFP had increased to 8189.0 ng/ml. Furthermore, an ileus occurred due to peritonitis carcinomatous. We therefore tried a regimen of 5-FU (500 mg/day via continuous infusion for 7 days) plus paclitaxel (120 mg weekly) at weekly intervals. After one treatment course, 5-FU administration was stopped but paclitaxel (120 mg) monotherapy was continued for 6 weeks. Serum AFP levels decreased again (to 709.7 ng/ml) and CT revealed that the liver metastases had either regressed or disappeared (Figure 3C). The patient currently has a very good quality of life, and is receiving weekly paclitaxel monotherapy as an outpatient. Three months have passed since 5-FU/paclitaxel treatment was started; no progression of liver metastases has been observed to date.

## Discussion

AFP-producing gastric cancers should be divided in to three subtypes: 1) hepatoid type; 2) yolk sac tumor-like type; and 3) fetal gastrointestinal type [13]. The hepatoid type was the most common one of AFP-producing gastric cancer, and they were described as hepatoid adenocarcinoma that means primary gastric cancer that are characterized by both hepatoid differentiation and the production of AFP [14,15]. Our case had the tumor that was classified as having a hepatoid subtype. Unfortunately, most hepatoid type tumor tends to be highly malignant [13]. AFP-producing gastric cancer has a high proliferative activity, weak apoptosis, and rich neovascularization [4]. Recent reports described that some factors associated with mitosis, cell movement, proliferative activity, and tumor progression such as Ki-67, hepatocyte growth factor (HGF) and its receptor, c-Met, vascular endothelial growth factor (VEGF) and its isoform VEGF-C, were found to be highly expressed in AFP-producing gastric cancer and might contribute to the poor prognosis and drug resistance of this tumor [4,16,17].

Few successful treatment options exist for AFP-producing gastric cancer. While surgical resection is considered to be somewhat effective, approximately 50% of patients who undergo curative resection of the primary tumor eventually die due to recurrence, most of them with multiple liver metastases [18]. Indeed, AFP-producing gastric cancer is associated with a high incidence of multiple liver metastases, which render resection impossible. Moreover, AFP-producing gastric cancer is reported to respond poorly to a number of chemotherapy regimens. The use of various chemotherapeutic protocols that are active in other types of cancer have been reported, although their efficacy in gastric cancer is controversial [7-11]. Some investigators have reported that AFP-producing gastric cancer can be treated successfully with preoperative (i.e., neoadjuvant) combination chemotherapy with EPI, 5-FU, and leucovorin (LV) [19]. Kochi *et al., *have also reported the use of a combination chemotherapy regimen consisting of 5-FU, LV, ETP, and CDDP (designated as the FLEP regimen) for inoperable stage IV gastric cancer [20,21]. FLEP chemotherapy was more effective for stage IV AFP-producing gastric cancer than for stage IV non-AFP-producing gastric cancer, and it improved the prognosis of AFP-producing gastric cancer due to downstaging.

TS-1 has shown superior results in the treatment of gastric cancer both as monotherapy and in combination with other agents. In a phase II clinical trial in Japan, single administration of TS-1 yielded a higher response rate (46.5%, 60/129 cases), longer median survival time (MST; 8.1 months), and lower incidence of adverse events compared to historical controls (e.g., 5-FU, methotrexate + LV, CDDP). It has proven to be one of the most promising new anti neoplastic agents [22]. However, in poorly differentiated gastric cancer, the agents described above are only effective in a small proportion of patients. Several case reports have demonstrated the efficacy of TS-1 monotherapy against malignant ascites. The novel antimitotic agent paclitaxel inhibits cell division by promoting tubulin polymerization and stabilizing microtubules [23]. This agent has been used as second-line treatment in patients with gastric cancer, particularly for cases refractory to first-line drugs (i.e., CDDP, 5-FU) or who experience recurrence following surgery.

As monotherapy, paclitaxel has been shown to result in a response rate of 26% in gastric cancer patients with previously treated by surgery and 21% in those with prior chemotherapy. Remarkably, paclitaxel is the only monotherapy shown to increase MST to longer than 300 days in patients with advanced and recurrent gastric cancer, regardless of prior treatment history [24]. Recently, some cases responding to paclitaxel treatment were reported. Chiba *et al., *reported the case of AFP-producing hepatoid adenocarcinoma in association with Barrett's esophagus with multiple liver metastases responding to paclitaxel/CDDP [25]. Hirashima *et al., *reported the successful bi-weekly paclitaxel treatment of an AFP-producing gastric cancer [11], however, there was no English literature which described the efficacy of paclitaxel for AFP-producing gastric cancers.

In our patient, we first used TS-1 and paclitaxel combination chemotherapy. Initially, the patient's AFP levels dramatically decreased and CT showed regression of metastatic liver tumors; however, multiple new liver metastases appeared and serum AFP levels increased after 5 months. Furthermore, the patient suffered an ileus due to peritonitis carcinomatous. Consequentially, we used combination 5-FU and paclitaxel chemotherapy with subsequent paclitaxel monotherapy as second-line treatment. This regimen resulted in the disappearance or reduction in size of many liver metastases, and greatly improved the patient's quality of life. The most significant toxicity, leukopenia, was effectively controlled with granulocyte colony-stimulating factors.

## Conclusion

To our knowledge, this is the first case report in the English literature of an AFP-producing gastric cancer associated with multiple liver metastases that was successfully treated with paclitaxel. We consider this rare case to be of significant value with respect to the treatment of AFP-producing gastric cancer with multiple liver metastases. We feel that combining surgery with chemotherapy such as paclitaxel will lead to a better prognosis in such cases. We also believe that our protocol is an effective and safe treatment for liver metastases associated with AFP-producing gastric cancer. Nevertheless, further clinical evaluation of this regimen in advanced AFP-producing gastric cancer with liver metastases is required.

## Competing interests

The author(s) declare that they have no competing interests.

## Authors' contributions

HT, HS, HT, NO, and TM conducted the clinical examination and surgery. TW, MS, YO, HF, AY, and YA performed pathological analysis. HT, HS, NO, and AY participated in the design of the study. TM conceived of the study, and participated in its design and coordination.

All authors read and approved the final manuscript.
